# Early sepsis does not stimulate reactive oxygen species production and does not reduce cardiac function despite an increased inflammation status

**DOI:** 10.14814/phy2.13231

**Published:** 2017-07-12

**Authors:** Thibault Léger, Alice Charrier, Clarisse Moreau, Isabelle Hininger‐Favier, Evangelia Mourmoura, Jean‐Paul Rigaudière, Elodie Pitois, Damien Bouvier, Vincent Sapin, Bruno Pereira, Kasra Azarnoush, Luc Demaison

**Affiliations:** ^1^ INRA, UMR 1019 Nutrition Humaine Clermont‐Ferrand Cedex 1 France; ^2^ Laboratoire de Bioénergétique Fondamentale et Appliquée INSERM U1055 Université Joseph Fourier Grenoble France; ^3^ Department of Medical Biochemistry and Molecular Biology CHU Clermont‐Ferrand Clermont‐Ferrand France; ^4^ Department of Clinical Research and Innovation CHU Clermont‐Ferrand Clermont‐Ferrand France; ^5^ Heart Surgery Department G. Montpied Hospital Clermont‐Ferrand University Hospital Clermont‐Ferrand France

**Keywords:** Contractility, heart, oxidative stress, sepsis

## Abstract

If it is sustained for several days, sepsis can trigger severe abnormalities of cardiac function which leads to death in 50% of cases. This probably occurs through activation of toll‐like receptor‐9 by bacterial lipopolysaccharides and overproduction of proinflammatory cytokines such as TNF‐*α* and IL‐1*β*. In contrast, early sepsis is characterized by the development of tachycardia. This study aimed at determining the early changes in the cardiac function during sepsis and at finding the mechanism responsible for the observed changes. Sixty male Wistar rats were randomly assigned to two groups, the first one being made septic by cecal ligation and puncture (sepsis group) and the second one being subjected to the same surgery without cecal ligation and puncture (sham‐operated group). The cardiac function was assessed in vivo and ex vivo in standard conditions. Several parameters involved in the oxidative stress and inflammation were determined in the plasma and heart. As evidenced by the plasma level of TNF‐*α* and gene expression of IL‐1*β* and TNF‐*α* in the heart, inflammation was developed in the sepsis group. The cardiac function was also slightly stimulated by sepsis in the in vivo and ex vivo situations. This was associated with unchanged levels of oxidative stress, but several parameters indicated a lower cardiac production of reactive oxygen species in the septic group. In conclusion, despite the development of inflammation, early sepsis did not increase reactive oxygen species production and did not reduce myocardial function. The depressant effect of TNF‐*α* and IL‐1*β* on the cardiac function is known to occur at very high concentrations. The influence of low‐ to moderate‐grade inflammation on the myocardial mechanical behavior must thus be revisited.

## Introduction

Sepsis, a general infection of the organism characterized by “blood poisoning”, is mainly due to bacterial contaminations. It strikes 30 million people worldwide every year and it is in constant progression in contrast with other pathologies such as cancer and cardiovascular diseases, whose incidence rates remain stable over time. Despite the medical improvements in the most developed countries, 30% of patients die from this pathology. Infants, elderlies, and people with deficient immune system (diabetes, AIDS, cancer, kidney, and liver diseases) are the most vulnerable. Severe sepsis is extremely expensive for health systems. Indeed, the duration of hospitalization is 2–3 weeks and is often followed by organ failure, which must be treated. A study performed by the Interpharma laboratory has been carried out in Switzerland in 2001 in order to estimate the costs linked to sepsis. In this study, Schmid et al. (2004) count approximately 10,000 cases of sepsis treated in Switzerland each year with 60 percent being severe sepsis. The costs for each patient reach 14,000 CHF, leading to global costs of 1 billion 400 million CHF. This amount of money includes only costs during hospitalization: the other costs resulting from possible complications and ambulatory cares are not taken into consideration. Moreover, the direct costs corresponding to the costs triggered during the stay in care units depict only 30 percent of global costs. Indirect costs related to productivity loss (sick leave, early retirement, and premature death) reach 100,000 CHF per patient with 95% being due to premature death.

Severe sepsis induces a general inflammation of the organism, abnormal blood clotting and systemic hypotension (Schouten et al. 2008). In most severe cases, when the hypotension is not resuscitated with crystalloid solution perfusion, the illness provokes a septic shock, which generally leads to death. Sepsis provokes multi‐organ ravages with the heart being responsible of death in 50% of cases (Smeding et al. 2012). The up‐to‐date measurement techniques of cardiac function (echography, MRI) do not reveal important modifications of this parameter during sepsis, except a decrease in diastolic function followed by a reduction of systolic function in the sickest patients (Hunter and Doddi 2010; Flynn et al. 2010). The most evident abnormality of the cardiovascular function is a decrease in arterial pressure associated with an initial compensatory stimulation of the heart rate (Cheung et al. 2015). The activation of the cardiac pump has for a long period been made responsible for the exhaustion of cardiomyocytes and the cardiac failure associated with the septic shock. Moreover, the systemic hypotension and associated increase in plasma fibrinogen (reduction of blood fluidity) suggests organ hypo‐perfusion and ischemia as the cause of the multi‐organ ravages. This hypothesis has however been recently modulated by the study of Antonucci et al. (2014) who demonstrated area of local ischemia in the myocardium of septic patients especially when the hypotension was severe. A more recent hypothesis has however emerged: bacterial lipopolysaccharides inhibit the contractility of isolated cardiomyocytes through a stimulation of the NFκB pathway (Avlas et al. 2011; Hobai et al. 2015). In polymicrobial sepsis, this would occur through a stimulation of Toll‐like receptor 9 which is known to trigger cardiac failure (Lohner et al. 2013) and death (Plitas et al. 2008). The phenomenon occurs through cellular death (Víctor et al. 2009). According to the literature, sepsis‐related cardiac disorders are due to the production of proinflammatory cytokines that are known to depress cardiac function (Ferrari 1999; Bujak and Frangogiannis 2009). However, this effect is observed only at very high cytokine concentrations and may not have clinical significance (Ferrari 1999). Moreover, cardiac mitochondria are important in the etiology of sepsis. Indeed, this pathology triggers a reduction of the oxidative phosphorylation associated with an important oxidative stress detected through a decrease in the aconitase/fumarase ratio (Mason and Stofan 2008). This is however in contradiction with the initial sepsis‐induced stimulation of the heart rate, since more energy is necessary to satisfy the needs of the cardiomyocytes.

This study aimed at determining the changes in the cardiac function during the early phase of sepsis in relation with the inflammatory status. We also investigated whether this was associated with an oxidative stress in plasma and heart. To perform this study, sepsis was induced in the rat by cecal ligation and puncture and it was maintained for 24 h. The cardiac function was then determined in vivo with a Millar pressure gauge inserted into the cavity of the left ventricle and ex vivo in the isolated heart perfused in standard conditions. The inflammatory status was determined in the systemic and myocardial compartments by evaluating the amount of TNF‐*α* by ELISA and the cardiac levels of TNF‐*α* and IL‐1*β* mRNAs, respectively. The oxidative stress, measured in the same compartments, was determined through the evaluation of protein thiol residues, total antioxidant defenses (FRAP), glutathione peroxidase activity and GSH to GSSG ratio.

## Methods

### Ethical approval

All experiments followed the European Union recommendations concerning the care and use of laboratory animals for experimental and scientific purposes. All animal work was approved by the local board of ethics for animal experimentation (Comité d'éthique pour l'expérimentation animale Auvergne) and notified to the research animal facility of our laboratory (authorization n° APAFIS#2213‐2016082409264678 v2).

### Experimental animals and surgical procedure

Sixty male Wistar rats (Janvier, Le Genest Saint Isle, France) were housed two per cage in our animal facility at 4 months of age. Half of them were subjected to cecal ligation and puncture in order to induce severe sepsis and the other half was sham‐operated.

Cecal ligation and puncture were performed according to Toscano et al. (2011). Briefly, the animals were anesthetized with isoflurane (induction 4%, maintenance 2%). After shaving the fur, the external abdominal wall was disinfected with alcoholic betadine and a horizontal incision was performed in the wall at the level of the cecum in order to reach the abdominal cavity and expose the cecum. A ligation of the cecum was carried out at 1.5 cm of the apex of the organ. Two perforations were performed through the cecum wall at 1 cm interval on the upper face of the organ with a 20 gauge needle and a soft pressure was applied on the cecum in order to facilitate externalization of digestive matter at the outer surface of the organ. The cecum was then returned into the abdominal cavity and buprenorphine (0.05 mg/kg body weight) was injected subcutaneously in the neck. The peritoneum was closed with 6.0 silk sutures and the skin with metal clips. Sham‐operated animals were treated identically as compared to septic animals except that the cecum was not subjected to ligation and puncture. After the surgery, isoflurane anesthesia was stopped and the rats were placed one per cage in the animal facility. The waking occurred after 3 to 5 min and rats were maintained in their cage during 24 h. One rat died during anesthesia and two septic animals died during the 24 h post‐surgery period.

### Evaluation of body composition

The animal body weight and body composition were determined just before the surgical procedure and at the end of the 24 h post‐operative period. Body composition was evaluated by nuclear magnetic resonance using an adequate spectrometer (EchoMRI LL, Houston, TX). The system allowed the estimation of fat, adipose and aqueous masses in living animals. At the end of the 24 h post‐operative period, the animals were sacrificed and the weights of hind leg muscles as well as those of abdominal and epididymal fats were measured. Abdominal fat was calculated as the sum of visceral and peri‐renal fats.

### Estimation of in vivo cardiac function

In vivo cardiac function was determined using a Millar pressure gauge (Harvard Apparatus, Les Ulis, France) introduced into the left ventricle. After anesthesia with ketamine (100 mg/kg) and xylazine (20 mg/kg) and heparinization of the rats (500 IU/kg), the gauge was introduced into the right carotid artery and immediately inserted into the left ventricle cavity. After a 10‐min period allowing stabilization of cardiac function, the left ventricle developed pressure, dP/dt_max_, dP/dt_min_ and heart rate were monitored. The aortic systolic, diastolic, and mean pressures were then determined by inserting the gauge into the aorta. The presented data represent the means of 100 cardiac cycles per rat for 30 rats per group. After the removal of the gauge, the right carotid was bound. Blood was collected from the abdominal aorta and centrifuged (1800 g, 10 min, 4°C) for plasma preparation. Once obtained, the plasma was distributed in different tubes, immediately frozen at the temperature of liquid nitrogen and stored at −80°C until different biochemical determinations were performed.

### Determination of ex vivo cardiac function

Cardiac function was estimated using the non‐recirculating Langendorff's method in standardized conditions. Briefly, just after estimation of the in vivo cardiac function and blood collection, the heart was rapidly collected and placed in cold (4°C) saline buffer until cessation of beating. It was then immediately perfused by the aorta at 37°C with a Krebs‐Heinselett buffer composed of (in mmol/L) NaCl (119), KCl (4,8), MgSO_4_ (1,6), NaHCO_3_ (22), KH_2_PO_4_ (1,2), CaCl_2_ (1,8), D‐glucose (11) and sodium hexanoate (0.5), pH 7.4. The perfusion buffer was constantly oxygenated with carbogen (95% O_2_–5% CO_2_). The perfusion was carried out within the 1st min after heart collection in order to avoid problems of cellular damages and preconditioning. The pulmonary artery was then cannulated for collection of coronary effluents. The heart was electrically paced from the 5th min to the end of perfusion at a rate of 370 beats/min. The perfusion flow was progressively raised at a rate of 12 mL/min during the first 5 min of perfusion and kept constant until the 30th min of perfusion. At the 30th min of perfusion, the perfusion flow was fixed at a value of 24 mL/min in order to maximally activate cardiac function and it was kept constant until the 40th min of perfusion. At the beginning of perfusion, a latex balloon related to a pressure gauge and an amplifier was inserted into the left ventricle. It was inflated until the diastolic pressure reached 10 mmHg. This allowed the evaluations of systolic, diastolic, and developed pressure as well as dP/dt_max_, dP/dt_min_ and heart rate. The rate pressure product was the product between heart rate and left ventricular developed pressure. The use of perfectly standardized perfusion conditions allowed the evaluation of the contractility and relaxation through the measurements of dP/dt_max_ and dP/dt_min_, respectively. A pressure gauge was also inserted just before the aortic cannula in order to estimate the perfusion pressure. In conditions of perfusion at fixed coronary flow, the perfusion pressure corresponds to the coronary pressure and changes in coronary pressure reveals modifications of coronary volume (coronary dilatation, coronary constriction and/or obliteration of coronary micro‐vessels). The parameters of cardiac function were evaluated at the 30th min of perfusion (coronary flow = 12 mL/min), at the 34th min of perfusion (coronary flow = 24 mL/min when the perfusion fluid reached temperature stabilization) and at the 40th min of perfusion (coronary flow = 24 mL/min). All the parameters of cardiac function were recorded and analyzed with a computer using the HSE software (Hugo Sachs Elektronik, March‐Hugstetten, Germany). The linear correlations between the metabolic efficiency and perfusion pressure as well as those between the dP/dt_max_ and perfusion pressure were calculated at each fixed coronary flow (12 and 24 mL/min). At the 30th and 40th min of perfusion, samples of arterial and venous perfusion fluid were anaerobically collected for determination of oxygen partial pressures with a blood gas analyzer (Radiometer, Neuilly‐Plaisance, France). Cardiac oxygen consumption was determined by subtracting the venous oxygen partial pressure to the arterial one and by multiplying the difference by the coronary flow. The cardiac metabolic efficiency was calculated as the ratio between the rate pressure product and the oxygen consumption.

### Gene expression

Gene expression of interleukin‐1*β* (IL‐1*β*) and tumor necrosis factor‐*α* (TNF‐*α*) were measured in cardiac homogenates. RNA extraction was performed using TRIzol^®^ (Thermo Scientific) according to the manufacturer's instructions. Chloroform was added (0.2 mL/mL of TRIzol^®^), and samples were mixed and centrifuged for 15 min at 12,000 g and 4°C. Aqueous phase containing RNA was collected, mixed with isopropanol to precipitate RNA, and centrifuged (12,000*g*, 4°C, 15 min). After centrifugation, the pellet was washed with ethanol 70% (v/v), dried, and suspended in water. RNA quantification and integrity were verified by measuring the ratio of optical density at 260 and 280 nm and by agarose gel migration, respectively. Two micrograms of total RNA was used for reverse transcription. The products of reverse transcription were used for reverse transcription quantitative polymerase chain reaction (RT‐qPCR) to evaluate gene expression. TaqMan low density array was used using 384‐well format plates on a 7900HT Fast Real Time PCR system (Applied Biosystems). Gene expression was performed using specific primers (sequences available on request) and Rotor‐Gene SYBR Green PCR master mix on a Rotor‐Gene Q System (Qiagen, Courtaboeuf, France). mRNA quantification was assayed using the ddCT method. *β*‐actin was used as the housekeeping gene.

### Other biochemical determinations

Plasma TNF‐*α* and triglycerides concentrations were determined using commercially available kits (ThermoFisher, Courtaboeuf, France). Plasma creatinine was determined spectrophotometrically using an enzymatic assay kit (Sigma‐Aldrich, L'Isle d'Abeau, France). Several parameters of the oxidative stress (protein thiol residues, total antioxidant defenses (FRAP), glutathione peroxidase activity, GSH and GSSG) were determined as already described previously (Mourmoura et al. 2013).

### Statistical analyses

Results are presented as mean ± S.E.M. The morphological and biochemical data as well as the results of the in vivo cardiac function were contrasted across the two groups by one‐way analysis of variance (ANOVA). Measures related to the ex vivo cardiac function were treated with repeated‐measures ANOVA to test the effect of sepsis (external factor), that of the perfusion time (internal factor) and their interaction (heart as subject factor). When required, group means were contrasted with a Fisher's LSD test.

Analysis of the correlations between the different parameters of the isolated perfused heart were two‐sided, with a type I error set at *α *= 0.05. Quantitative data were presented as the mean ± standard deviation (SD) or the median (interquartile range) according to statistical distribution (assumption of normality assessed by using the Shapiro‐Wilk test). The relationships between quantitative data were studied using correlation coefficients (Pearson or Spearman according to statistical distribution). Then, to take into account between and within subject variability (due to several measures for a same animal), random‐effects for correlated measures were performed rather than usual statistical tests, which would not be appropriate due to the fact that the hypothesis of independence data was not verified. A probability (*P*) less than 0.05 was considered significant. The statistical analysis was performed using Stata software, version 13 (StataCorp, College Station, TX, US).

## Results

### Inflammation status

**Figure 1:**
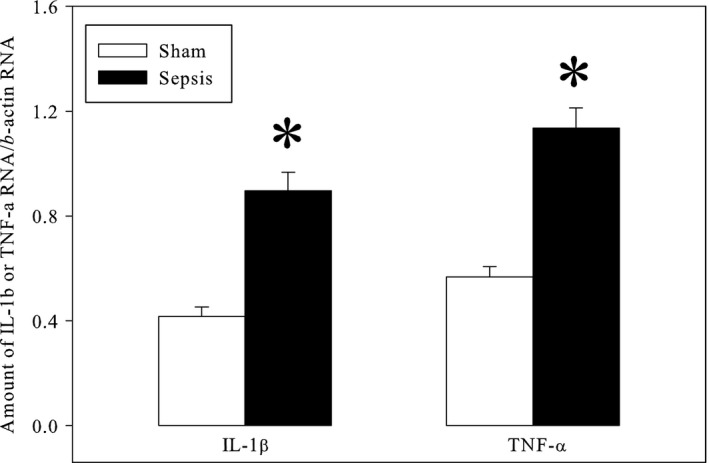
Gene expression of IL‐1β and TNF‐*a* in the perfused heart. The results represent the means and S.E.M. of 30 animals per group. Sham: sham‐operated animals; Sepsis: septic animals. *: significantly different.

The level of TNF‐*α* was increased from 10.8 ± 0.9 in the sham group to 20.4 ± 5.2 pg/mL in the sepsis group (*P* < 0.05), confirming the development of an inflammatory reaction in the sepsis group. The septic heart also displayed an increased inflammation status (Fig. 1) with raised mRNAs for TNF‐*α* (+115%, *P* < 0.001) and IL‐1*β* (+100%, *P* < 0.001) even after the ex vivo perfusion. Plasma triglycerides were also increased (0.198 ± 0.010 vs. 0.333 ± 0.016 g/L in the sham‐operated and sepsis groups, *P* < 0.001).

### Plasma and cardiac oxidative stress

Despite the sepsis‐induced augmentation of the inflammatory status, the plasma and heart oxidative stress were not increased (Table 1). Plasma glutathione oxidase activity was reduced by sepsis (−11%) and the myocardial GSH to GSSG ratio was increased (+34%), suggesting that the production of reactive oxygen species was reduced in these two compartments.

**Table 1:** Plasma and cardiac oxidative stress

Organ	Biochemical parameters	Sham	Sepsis
Plasma	Thiols (*μ*moles/gprot)	2.84 ± 0.07	2.98 ± 0.07
	FRAP (*μ*moles/gprot)	7.2 ± 0.2	7.4 ± 0.4
	GPX (U/gprot)	137 ± 3	122 ± 3^a^
Heart	Thiols (*μ*moles/gprot)	84 ± 3	84 ± 3
	FRAP (*μ*moles/gprot)	118 ± 4	116 ± 4
	GPX (U/gprot)	1476 ± 32	1434 ± 25
	GSH (*μ*moles/gprot)	120 ± 3	120 ± 2
	GSSG (*μ*moles/gprot)	2.2 ± 0.2	1.6 ± 0.2
	GSH/GSSG	67 ± 6	90 ± 7^a^

The results represent a mean and S.E.M. of 30 animals in each group. Sham, sham‐operated animals; Sepsis, septicemic animals; FRAP, total antioxidant activity; GPX, glutathione peroxidase activity; GSH, reduced glutathione; GSSG, oxidized glutathione; gprot, g of proteins; U, unit.

a Significantly different.

### Morphological data

The animal weights as well as fat, lean, and aqueous masses were similar before and 24 h after the surgical procedure (Table 2). The evaluations of organ weights were performed only 24 h after the surgery since such determinations necessitated animal sacrifice. The weights of peri‐renal, visceral, abdominal and epididymal adipose tissues as well as those of the different hind leg muscles (quadriceps, extensor digitorus longus, gastrocnemius, plantaris, soleus and tibialis) and heart were not modulated by sepsis.

**Table 2:** Morphological characteristics of the animals

	Sham		Sepsis	
	Pre‐op.	Post‐op.	Pre‐op.	Post‐op.
Animal weight (g)	575 ± 13	559 ± 13	564 ± 10	552 ± 10
Fat mass (g/100 g bw)	14.3 ± 0.6	14.1 ± 0.6	14.1 ± 0.6	14.3 ± 0.7
Lean mass (g/100 g bw)	77.4 ± 0.6	77.5 ± 0.6	77.6 ± 0.6	76.2 ± 0.7
Aqueous mass (g/100 gbw)	55.7 ± 0.5	56.1 ± 0.5	55.7 ± 0.5	55.9 ± 0.5
Peri‐renal AT (g/100 g bw)	nd	1.41 ± 0.06	nd	1.45 ± 0.06
Visceral AT (g/100 g bw)	nd	1.68 ± 0.06	nd	1.86 ± 0.08
Abdominal AT (g/100 g bw)	nd	3.11 ± 0.11	nd	3.28 ± 0.13
Epididymal AT (g/100 g bw)	nd	1.33 ± 0.05	nd	1.40 ± 0.05
Quadriceps (mg/100 g bw)	nd	609 ± 20	nd	610 ± 16
EDL (mg/100 g bw)	nd	41.3 ± 0.6	nd	39.6 ± 0.7
Gastrocnemius (mg/100 g bw)	nd	428 ± 6	nd	436 ± 7
Plantaris (mg/100 g bw)	nd	88.1 ± 1.7	nd	86.7 ± 2.3
Soleus (mg/100 g bw)	nd	44.3 ± 1.4	nd	43.3 ± 1.4
Tibialis (mg/100 g bw)	nd	151 ± 2	nd	148 ± 2
Hind leg muscles (mg/100 g bw)	nd	769 ± 10	nd	760 ± 12
Heart (mg dw/100 g bw)	nd	58 ± 1	nd	58 ± 2

The results represent the means and S.E.M. of 30 animals per group. Sham, sham‐operated animals; Sepsis, septicemic animals; Pre‐op., pre‐operative situation; Post‐op., post‐operative situation 24 h after the surgery; dw, dry weight; bw, body weight; AT, adipose tissue; EDL, extensor digitorus longus; nd, not determined since the weight was not measurable without sacrificing the animals. No significant differences were observed for all the data.

**Figure 2:**
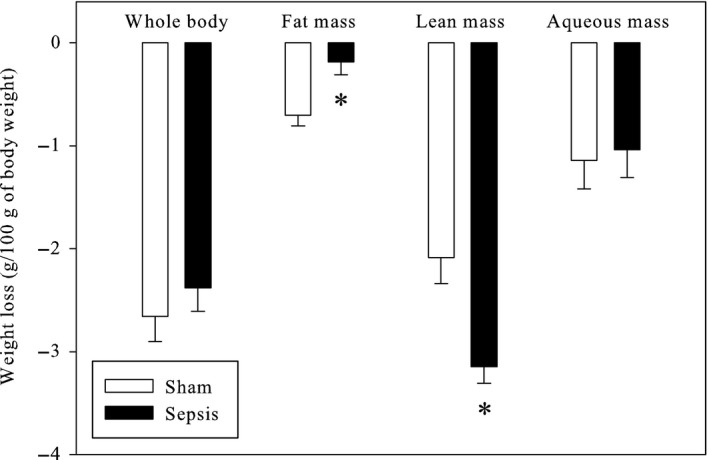
Body mass changes induced by the sham‐operation and cecal ligature and puncture in rodents. The results represent the means and S.E.M. of 30 animals per group. Sham: sham‐operated animals; Sepsis: septic animals. *: significantly different.

The surgery‐induced changes in body composition are presented in Figure 2. Sepsis tended to reduce the loss of animal weight, but the difference was not significant. The sham‐operation induced a significant increase in fat mass disappearance compared to sepsis (+276%). In contrast, sepsis favored the loss of lean mass (+51%) compared to sham‐operation. The loss of aqueous mass was not affected by the type of surgery.

### In vivo cardiac function

Evaluation of aortic pressures (Table 3) shows that sepsis tended to decrease the aortic systolic, diastolic and mean pressures, but the differences did not reach significance. The left ventricle developed pressure, contractility (dP/dt_max_) and relaxation (dP/dt_min_) were not modulated by sepsis. Conversely, the heart rate was significantly augmented (+11%).

**Table 3:** Cardiovascular parameters in the in vivo situation

Parameter	Sham	Sepsis
Aortic systolic pressure (mmHg)	91 ± 3	84 ± 2
Aortic diastolic pressure (mmHg)	63 ± 3	56 ± 3
Mean aortic pressure (mmHg)	76 ± 3	70 ± 2
Left ventricle developed pressure (mmHg)	104 ± 3	96 ± 2
Heart rate (beats/min)	246 ± 5	273 ± 7^a^
dP/dt_max_ (mmHg/s)	5279 ± 212	5268 ± 288
dP/dt_min_ (mmHg/s)	4342 ± 235	4148 ± 193

The results represent a mean and S.E.M. of 30 animals in each group. Sham, sham‐operated animals; Sepsis, septicemic animals; dP/dt max, contraction; dP/dt min, relaxation.

a Significantly different.

### Ex vivo cardiac function

**Figure 3:**
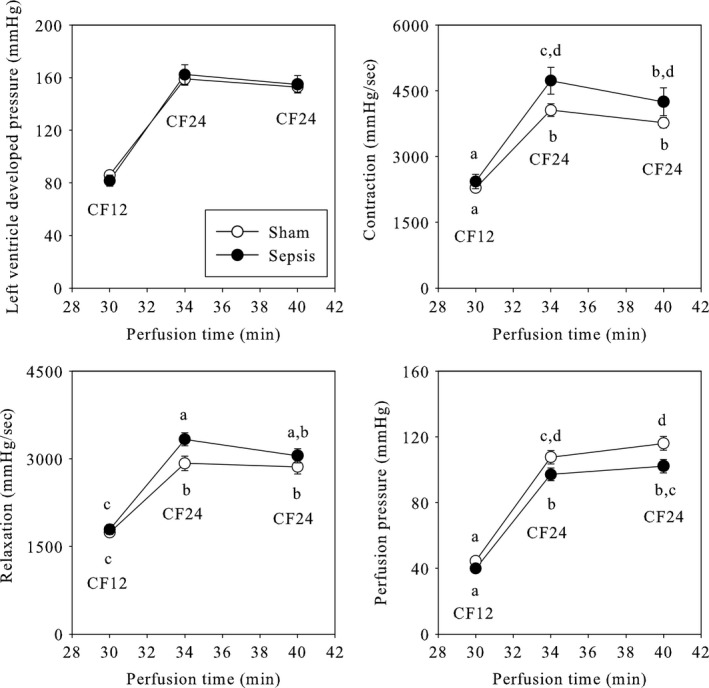
Cardiac mechanical function in ex vivo standardized conditions. The results represent the means and S.E.M. of 30 animals per group. Sham: sham‐operated animals; Sepsis: septic animals; CF12 and CF24: coronary flow fixed at the value of 12 or 24 mL/min, respectively. a, b, c, d: a mean without a common letter in a same panel indicate a significant difference.

The function of the isolated hearts was evaluated in standard conditions (Fig. 3) at two different fixed coronary flows (12 mL/min until the 30th min and 24 mL/min from the 30th to the 40th min of perfusion). The left ventricle developed pressure, whose changes were similar to those of the rate pressure product since all the hearts were electrically paced at a rate of 370 beats/min, was strongly increased (+88%) by the augmentation of coronary flow. Similar results were observed for contractility (+78%), relaxation (+73%) and coronary pressure (+151%).

Sepsis did not alter the left ventricle developed pressure compared to the sham‐operation whatever the fixed coronary flow. This was not the case for contractility. Indeed, sepsis increased the value of this parameter at the 34th min of perfusion (+17%) at a coronary flow rate of 24 mL/min. However, the difference disappeared for a longest perfusion period. A similar pattern was observed for relaxation with a + 14% increase at the 34th min of perfusion. However, this was different for the perfusion pressure. Similar when the flow was fixed at 12 mL/min, this parameter was decreased by sepsis for the highest coronary flow (−10 and −12% at the 34th and 40th min of perfusion). This occurred while the heart dry weight was unchanged by sepsis (318 ± 6 and 321 ± 7 mg for the sham and sepsis groups).

**Figure 4:**
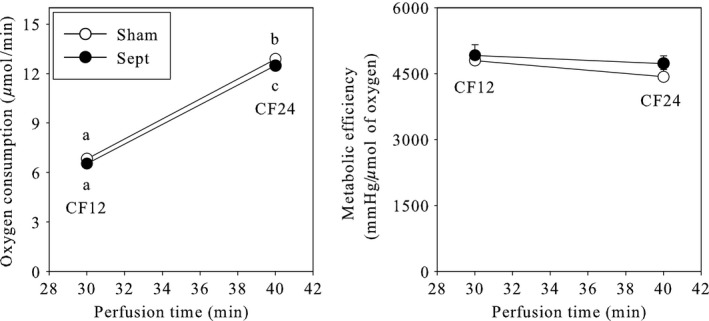
Cardiac oxidative metabolism in ex vivo standardized conditions. The results represent the means and S.E.M. of 30 animals per group. Sham: sham‐operated animals; Sepsis: septic animals; CF12 and CF24: coronary flow fixed at the value of 12 or 24 mL/min, respectively. a, b, c: a mean without a common letter in a same panel indicate a significant difference.

Myocardial oxygen consumption was also determined at the 30th and 40th min of perfusion (Fig. 4). Following the stimulation of cardiac function, the oxygen consumption was increased by the augmentation of coronary flow (+90%). No sepsis‐induced difference was observed at the lowest coronary flow, but a slight but significant reduction (‐3%) was noticed at 24 mL/min. However, the cardiac metabolic efficiency (ratio between the rate pressure product and the oxygen consumption) was never significantly altered.

### Relationships between different parameters of the ex vivo cardiac function

**Figure 5:**
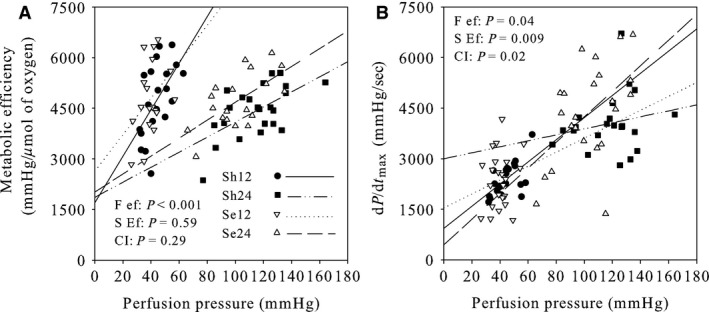
Correlations between the perfusion pressure and metabolic efficiency (panel A) as well as the perfusion pressure and dP/dt_max_ (panel B) of the isolated perfused hearts. The linear regression lines for panel A are: ME = 69 PPERF + 1692 (*P* < 0.001), ME = 22.3 PPERF + 1861 (*P* < 0.001), ME = 54.12 PPERF + 2630 (*P* = 0.07) and ME = 2658 PPERF + 2014 (*P* < 0.001) with 0.47, 0.21, 0.14 and 0.54 for correlation coefficients for the Sh12, Sh24, Se12 and Se24 groups. ME and PPERF are the metabolic efficiency and perfusion pressure. Those for panel B are: MAX = 21.45 PPERF +1469 (*P* = 0.201), MAX = 13.74 PPERF + 2514 (*P* = 0.213), MAX = 23.34 PPERF + 1469 (*P* = 0.141) and MAX = 41.84 PPERF + 77 (*P* = 0.002) with 0.63, 0.51, 0.34 and 0.55 for correlation coefficients where MAX is the dP/dt_max_. Sh12: hearts of sham‐operated animals perfused at a flow rate of 12 mL/min; Sh24: hearts of sham‐operated animals perfused at a flow rate of 24 mL/min; Se12: hearts of septic animals perfused at a flow rate of 12 mL/min; Sh12: hearts of septic animals perfused at a flow rate of 12 mL/min; F ef: flow effect; S ef: sepsis effect; CI: cross‐interaction.

The relationships between the perfusion pressure and metabolic efficiency for the four groups studied are presented in Figure 5A. Although the slopes of the linear regression lines were reduced when the coronary flow was increased from 12 to 24 mL/min (−60%), the cecal ligation and puncture did not affect these parameters. The cross‐interaction between the effects of the coronary flow and sepsis was not significant.

The correlations linking the perfusion pressure with the dP/dt_max_ (contractility) are shown in Figure 5B. The slopes of the lines were significantly increased when the coronary flow rate was augmented to 24 mL/min (+24%) and when sepsis was induced (+85%). Interestingly, a significant cross‐interaction was noticed. When the coronary flow rate was fixed at 12 mL/min, the line was less steep in the sepsis group, but when the flow was increased to 24 mL/min, the slope was strongly increased by the pathology (+205%) whereas that of the sham‐operated group was reduced.

### Renal function

**Figure 6:**
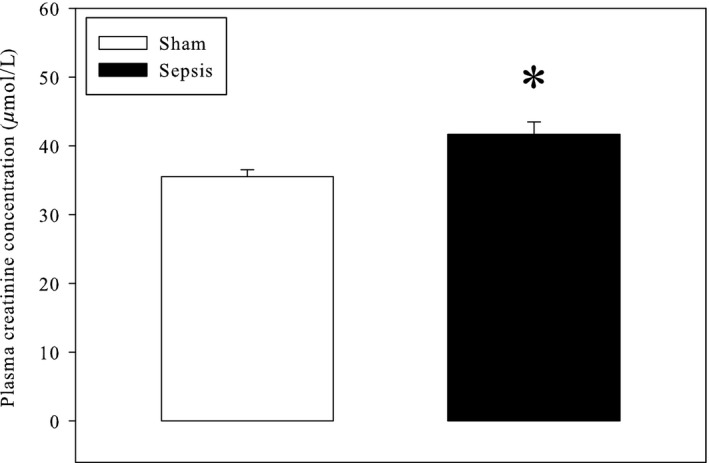
Plasma creatinine levels. The results represent the means and S.E.M. of 30 animals per group. Sham: sham‐operated animals; Sepsis: septic animals. *: significantly different.

Plasma creatinine was increased by sepsis (+17%, Fig. 6).

## Discussion

This study aimed at determining the changes in the cardiac function during the early phase of sepsis in relationship with the inflammatory status and at investigating whether it is associated with an oxidative stress in plasma and heart. The cardiac function was evaluated in the in vivo situation, but also ex vivo in a model of isolated rat heart perfused in standard conditions. The cecal ligation and puncture augmented the inflammatory status of the plasma and heart as evidenced by the increased amount of TNF‐*α* in the plasma and the increased gene expression of TNF‐*α* and IL‐1*β* in the myocardium. However, this did not increase the oxidative stress of the two compartments. On the contrary, this last parameter was reduced as suggested by the reduced glutathione oxidase activity of the plasma and the augmented GSH to GSSG ratio of the heart. All these modifications were associated with an activation of the heart in the in vivo and ex vivo situations.

### Limitations of the study

In our study, the body composition of the animals was not modified by the cecal ligation and puncture (CLP) in comparison with the sham group. The duration of the post‐surgery period (1 day) was probably too short in order to allow any significant change in the fat and lean masses. However, another factor played an important role: the sham‐operated animals were deeply affected by their surgery (opening of the abdominal wall and externalization of the cecum without ligation and puncture). During the post‐surgery period, these animals did not eat and drink a lot (data not shown), which led to a small and not significant loss of body weight of similar amplitude as that estimated in the sepsis group (−2.8 and −2.1 g/100 g of body weight in the sham‐operated and sepsis group, respectively). Injection of lipopolysaccharides or living bacteria versus vehicle would have been another alternative in order to better visualize the effect of sepsis on the body composition, but the cecal ligation and puncture remains better to simulate an in vivo situation. An important result of the body composition confirmed the development of sepsis in our septic group: the loss of lean mass was significantly ampler compared with the sham‐operated group, suggesting cachexia, which is supposed to occur during sepsis (Londhe and Guttridge 2015). Furthermore, the renal function was damaged as evidenced by the increased concentration of plasma creatinine. The colon ascendens stent peritonitis (CASP) is also an interesting model to simulate an in vivo situation: it allows a constant delivery of bacteria into the abdominal cavity in contrast with the CLP model which is more transitory on this viewpoint. CASP is thus more severe than CLP with a high inflammatory reaction (Maier et al. 2004). However, it is less adapted to the study of the hyperdynamic phase of sepsis and its consequences on the cardiac function and it was thus rejected of our experimental design.

Another limitation of our study lies in the use of a particular model of isolated heart perfusion. In general, isolated hearts are perfused at fixed pressure (59–74 mmHg) against a column of water of constant height. In these conditions, the coronary flow is determined as a function of the coronary bed resistances. The flow decreases slightly but gradually with the perfusion time indicating a progressive increase in the coronary bed resistances. After a 30‐min perfusion, the flow generally reaches approximately 11–13 mL/min for the size of the hearts used in this study. This is why we decided to perfuse the heart at a fixed coronary flow of 12 mL/min. In our study, the nutritional fluid was constantly perfused at this rate with the aid of a peristaltic pump. At the beginning of the perfusion, the flow was gradually increased (during 5 min) to 12 mL/min in order to not destroy the endothelial cells. This is not the case for the perfusion at constant pressure via a column of water, since the coronary flow is abruptly increased to its maximum. Under these conditions, the coronary flow is very high. As measured with a flow gauge, it reaches values higher than 20 mL/min, which can alter endothelial cells and contribute to the progressive vasoconstriction encountered in this kind of perfusion. In our experiment, we implanted a pressure gauge near the aorta in order to measure the coronary pressure. We noticed that the coronary bed resistances did not increase during the 30‐min perfusion (data not shown) as it could have been expected by the decreased coronary flow with the perfusion at constant pressure. It is likely that the perfusion at a constant flow rate of 12 mL/min preserved the endothelial cells and prevented the vasoconstriction resulting from cell damages. However, the coronary pressures were low (45 ± 2 and 40 ± 2 mmHg for the sham and sepsis groups, respectively). In addition to maintaining the vascular cells healthy, this kind of perfusion allowed the upholding of a perfectly constant mechanical activity of the isolated heart during the 30‐min perfusion, which suggests that cardiomyocytes were also maintained in healthy conditions.

### Inflammation

Our results showed that cecal ligature and puncture increased the inflammatory status of the animals. It was observed in the systemic circulation through an augmented plasma level of TNF‐*α*. Bacterial lipopolysaccharides link to Toll‐like receptors (TLR) and activate the NFκB pathway. In cultured skeletal muscle cells, high doses of lipopolysaccharides (500 pmoles/mL) elicit the expression of several inflammatory cytokines such as IL‐6 and MCT‐1 (Frost et al. 2003; Frisard et al. 2010); lower doses (50 pmoles/mL) stimulate glucose oxidation and inhibit fatty acid degradation without triggering the expression of proinflammatory cytokines. These last conditions lead to the accumulation of triglycerides and lactate (Frisard et al. 2010) in the plasma of septic subjects. In our study, it is likely that skeletal muscle inflammation occurred, since the loss of lean mass was higher in the septic group compared to the sham‐operated animals. Indeed, inflammation is known to induce loss of skeletal muscle mass (Londhe and Guttridge 2015).

In the heart, we observed that the cecal ligation and puncture increased the expression of TNF‐*α* and IL‐1*β*. TNF‐*α* is the main cytokine synthesized during endotoxemia and is considered to be responsible for the expression of IL‐1*β*, IL‐2 and IL‐6 (Ferrari 1999). It has several cardio‐vascular effects: at high doses, it reduces myocardial contraction through a mechanism involving an increased nitric oxide production. It also triggers hypotension, reduced systemic vascular resistances and biventricular dilatation (Sedger and McDermott 2014). The depressant effect of TNF‐*α* on the cardiac mechanical function occurs through an inhibition of the L‐type calcium channels and calcium release by the sarcoplasmic reticulum (Ferrari 1999). However, it also happens through oxidative stress and cardiomyocytes apoptosis (Tartaglia et al. 1993). IL‐1*β* is also cardio‐depressant, pro‐apoptotic and triggers hypertrophy (Bujak and Frangogiannis 2009). It would also play a central role in sepsis‐induced contractile dysfunction.

### Cardiac mechanical function

Sepsis is usually known to depress cardiac function (Hunter and Doddi 2010). In humans, changes are difficult to estimate, since the measurements are performed in inactive patients whose cardiac activity is low. They are initiated by a decrease in diastolic function, a status that progresses toward systolic function reduction with the worsening of sepsis (Flynn et al. 2010). In our study, the sepsis that was performed did not deteriorate cardiac function. On the contrary, it slightly improved it. This was observed in vivo through an increased heart rate at similar rates of contraction and relaxation. The arterial pressures were slightly reduced by sepsis to a level which was however not significant. This could increase the heart rate and cardiac output by baroreflex resetting. However, an improved cardiac function was also noticed ex vivo when the heart rate of the isolated heart was fixed via electrical pacing and the stretching exerted on myocardial fibers was controlled by the latex balloon inflation and was similar for each heart. Under these circumstances, the contractility and relaxation were slightly improved. The differences did not appear when the cardiac mechanical work was low. However, the increase in coronary flow from 12 to 24 mL/min increased the cardiac work and exacerbated the sepsis‐induced changes of cardiac contractility and relaxation. These results were validated by the study of the correlations between perfusion pressure and contractility (dP/dt_max_). The correlation coefficients of the regression lines were statistically analyzed as functions of coronary flow and surgical procedure. A significant cross‐interaction was observed. For the low coronary flow (12 mL/min), the regression line for the sepsis group was less steep than that for the sham‐operated group. For the high coronary flow (24 mL/min), this was the contrary. The relation between the perfusion pressure and the contractility fits probably a Michaelis‐Menten curve. Our results show that the curves are shifted for the sepsis and sham‐operated groups. Interestingly, the curve seems to have reached a status near saturation for the sham‐operated group when the coronary flow was fixed at 24 mL/min. This was not the case for the sepsis group for which the slope continued to climb abruptly. The differences might be due to the changes in coronary pressure which did not reach obviously a maximum for the septic group. It suggests that with a coronary flow set at values higher than 24 mL/min, the differences in contractility between the two groups would have been even ampler. It appears thus difficult to know whether the sepsis‐induced increase in cardiac function observed in the in vivo situation was due only to a baroreflex resetting or to an increased intrinsic myocardial contractility. Both phenomena could be involved. However, the improved ex vivo cardiac function observed in our study reflects probably the fact that sepsis was of low severity.

Despite the increased cardiac mechanical work observed with sepsis at a high coronary flow, the oxygen consumption was reduced. Yet, the differences were not sufficient to improve significantly the cardiac metabolic efficiency (rate pressure product to oxygen consumption ratio). This result is confirmed by the fact that sepsis had no effect on the correlations between perfusion pressure and metabolic efficiency. Interestingly, the sepsis‐induced improvement of the cardiac mechanical function was associated with a significant reduction of the coronary perfusion pressure, suggesting the involvement of vasodilatation agents such as nitric oxide. Sepsis is known to stimulate the protein expression of the inducible form of nitric oxide (NO) synthase, which could increase cellular NO release.

The slightly improved cardiac work observed with sepsis in our study was associated with an increased inflammatory status of the circulation and heart. The high levels of TNF‐*α* and IL‐1*β* mRNAs should have depressed the cardiac mechanical activity (Schulz et al. 1995). This was not the case. We evaluated thus the oxidative stress in plasma and heart. In plasma, the amount of protein thiol residues was not modified by sepsis. The total antioxidant defenses (FRAP) were unchanged, but the glutathione peroxidase activity was decreased, suggesting that no oxidative stress occurred. Similarly, in the heart no oxidative stress occurred, but the high GSH/GSSG ratio suggests that less reactive oxygen species (ROS) were detoxified by the glutathione peroxidase. We were not able to evaluate the ROS production directly in our study, since measurements should have been done in the beating heart. One solution in order to estimate correctly the cardiac ROS production and mechanical function in parallel would have been to determine the release of ascorbyl radicals in the coronary effluents by electronic paramagnetic resonance, as we did it in the past (Demaison et al. 2001). However, this technology is no longer available for us. Three different mechanisms could explain the differences: (1) a stimulation of the cardiac mechanical function at the early phase of sepsis could be responsible for the lower ROS production. Indeed, a higher energy utilization by the contractile machinery necessitates a higher ATP production, an activation of the oxidative phosphorylation and a dissipation of the ΔΨ. Yet, it is well known that the lowest the ΔΨ, the lowest the mitochondrial ROS generation; (2) a lower ROS production not related to the increased cardiac mechanical function, but due to other organelles or enzymes. Albayrak et al. (2011) have shown that sepsis increases cardiac catalase and myeloperoxidase activities in the ovariectomized rat. This would increase the GSH to GSSG ratio and would preserve the cellular NADPH. Indeed, re‐reduction of GSSG to GSH is performed by the glutathione reductase which consumes NADPH. This would also preserve the NADH which is in equilibrium with NADPH. More NADH would thus be available for the energy synthesis through the oxidative phosphorylation and this would allow the stimulation of cardiac mechanical function. Ávila et al. (2016) reported that the productions of OH^.^ and H_2_O_2_ by isolated rat papillary muscle were increased and the contractility was reduced by increasing ferrous iron concentrations. This deleterious effect was reversed by catalase; (3) a stimulation of the NADH production through an activation of the intermediary metabolism which would feed both the oxidative phosphorylation and re‐reduction of GSSG. This would explain the increased mechanical work and GSH to GSSG ratio in spite of similar or even increased ROS production. However, it would also necessitate a stimulation of the oxidative metabolism. Yet, the cardiac oxygen consumption was reduced by the sepsis in our study. The last explanation is thus not plausible. Conversely, the first two explanations are possible and we can conclude that the observed changes in cardiac function were due to a lower ROS production. However, we are unable to know if the lower ROS production was due to the stimulation of the cardiac mechanical function and ΔΨ dissipation or to another mechanism independent of the oxidative phosphorylation. The two mechanisms could affect differently the NADH to NAD^+^ ratio. Unfortunately, we did not measure the NADH to NAD^+^ ratio in our study due to lack of biological material. Further studies in isolated mitochondria could help to better understand the observed effects.

The decreased ROS production is in complete contradiction with what is known about sepsis in the literature. Indeed, sepsis and inflammation are generally associated with oxidative stress (Huet et al. 2011). Septic cardiac mitochondria also display a high oxidative stress as evidenced by a low aconitase to fumarase ratio (Mason and Stofan 2008). In our study, we did not measure the protein expression of TNF‐*α* and IL‐1*β* in the heart: their level could be only slightly affected and the effect of a low‐intensity inflammation on this organ is not well documented. The fact that our septic heart displayed a lower ROS production despite the increased level of proinflammatory cytokine mRNAs might be due to the 40‐min perfusion protocol which possibly washed out the deleterious molecules present in the systemic circulation without succeeding in clearing the excess TNF‐*α* and IL‐1*β* mRNAs. The protein expression for these interleukins might be stopped by the perfusion itself. However, the increased cardiac function was also observed in vivo, indicating that the isolation and perfusion did not impact the behavior of the hearts. The effects on the parameters of the oxidative status also occurred in the in vivo situation as evidenced by the lower glutathione peroxidase activity necessary to maintain a similar oxidative stress in the plasma.

In conclusion, early sepsis triggered a slight stimulation of the cardiac mechanical function in the in vivo and ex vivo situations despite an increased inflammation status. The phenomenon was associated with a slight reduction of ROS production which can explain the vasodilatation of the coronary bed. However, our results cannot determine whether this reduced ROS production resulted from the activation of the mechanical activity and ΔΨ dissipation or from ΔΨ‐independent superoxide generation in various organelles and enzymes. To the best of our knowledge, it is the first time that sepsis has been associated with increased inflammation and reduced ROS production. This might be due to the fact that sepsis was at its early phase in our experiment and triggered only a low‐ to moderate‐grade inflammation. On the contrary, a more severe sepsis could further increase the inflammatory status, augment the oxidative stress and reduce the cardiac mechanical function. In any case, the effects of a low‐grade inflammation on the cardiac function should be revisited in order to determine whether it can affect beneficially the oxidative stress and cardiac function.

## Conflict of Interests

None declared.
